# Multi-omics analysis reveals key regulatory defense pathways and genes involved in salt tolerance of rose plants

**DOI:** 10.1093/hr/uhae068

**Published:** 2024-03-02

**Authors:** Haoran Ren, Wenjing Yang, Weikun Jing, Muhammad Owais Shahid, Yuming Liu, Xianhan Qiu, Patrick Choisy, Tao Xu, Nan Ma, Junping Gao, Xiaofeng Zhou

**Affiliations:** Beijing Key Laboratory of Development and Quality Control of Ornamental Crops, Department of Ornamental Horticulture, China Agricultural University, Beijing 100193, China; Beijing Key Laboratory of Development and Quality Control of Ornamental Crops, Department of Ornamental Horticulture, China Agricultural University, Beijing 100193, China; Flower Research Institute, Yunnan Academy of Agricultural Sciences, Kunming 650205, China; Beijing Key Laboratory of Development and Quality Control of Ornamental Crops, Department of Ornamental Horticulture, China Agricultural University, Beijing 100193, China; Beijing Key Laboratory of Development and Quality Control of Ornamental Crops, Department of Ornamental Horticulture, China Agricultural University, Beijing 100193, China; Beijing Key Laboratory of Development and Quality Control of Ornamental Crops, Department of Ornamental Horticulture, China Agricultural University, Beijing 100193, China; LVMH Recherche, 185 avenue de Verdun F-45800 St., Jean de Braye, France; LVMH Recherche, 185 avenue de Verdun F-45800 St., Jean de Braye, France; Beijing Key Laboratory of Development and Quality Control of Ornamental Crops, Department of Ornamental Horticulture, China Agricultural University, Beijing 100193, China; Beijing Key Laboratory of Development and Quality Control of Ornamental Crops, Department of Ornamental Horticulture, China Agricultural University, Beijing 100193, China; Beijing Key Laboratory of Development and Quality Control of Ornamental Crops, Department of Ornamental Horticulture, China Agricultural University, Beijing 100193, China

## Abstract

Salinity stress causes serious damage to crops worldwide, limiting plant production. However, the metabolic and molecular mechanisms underlying the response to salt stress in rose (*Rosa* spp.) remain poorly studied. We therefore performed a multi-omics investigation of *Rosa hybrida* cv. Jardin de Granville (JDG) and *Rosa damascena* Mill. (DMS) under salt stress to determine the mechanisms underlying rose adaptability to salinity stress. Salt treatment of both JDG and DMS led to the buildup of reactive oxygen species (H_2_O_2_). Palisade tissue was more severely damaged in DMS than in JDG, while the relative electrolyte permeability was lower and the soluble protein content was higher in JDG than in DMS. Metabolome profiling revealed significant alterations in phenolic acid, lipids, and flavonoid metabolite levels in JDG and DMS under salt stress. Proteome analysis identified enrichment of flavone and flavonol pathways in JDG under salt stress. RNA sequencing showed that salt stress influenced primary metabolism in DMS, whereas it substantially affected secondary metabolism in JDG. Integrating these datasets revealed that the phenylpropane pathway, especially the flavonoid pathway, is strongly enhanced in rose under salt stress. Consistent with this, weighted gene coexpression network analysis (WGCNA) identified the key regulatory gene *chalcone synthase 1* (*CHS1*), which is important in the phenylpropane pathway. Moreover, luciferase assays indicated that the bHLH74 transcription factor binds to the *CHS1* promoter to block its transcription. These results clarify the role of the phenylpropane pathway, especially flavonoid and flavonol metabolism, in the response to salt stress in rose.

## Introduction

Rose (*Rosa* spp.) is a popular ornamental crop that is also used in the cosmetics, perfume and medicine. Rose plants contains various bioactive substances, including flavonoids, fragrant components, and hydrolysable and condensed tannins, which have high value and market potential [1]. However, soil salinization is common in many rose-growing regions, and high salt concentrations in soil can severely inhibit rose plant growth, reduce flower quality, and cause significant economic losses [2]. Additionally, salt stress can enhance the secondary metabolites of roses such as citronellol, geraniol, and phenyl ethyl alcohol [3, 4]. Such alterations in secondary metabolites may help to regulate the salt tolerance of rose. Research on roses has focused mainly on flower quality, petal development, and flower bloom [5–7], and there are limited data available regarding signaling pathways linking plant development and secondary metabolites associated with salt stress.

In plants, salt stress induces osmotic imbalances, which lead to the closure of leaf stomata, limit photosynthesis, and affect plant growth and metabolism [8]. To alleviate osmotic stress and protect themselves from its adverse effects, plants accumulate numerous compatible solutes (such as soluble proteins, soluble sugars, and proline), known collectively as osmoprotectants [9]. Moreover, plants generate reactive oxygen species (ROS) to cope with salt stress [10]. Nevertheless, excessive ROS accumulation can lead to oxidative DNA damage, affect protein biosynthesis, and ultimately result in cell damage and death [11, 12]. Plant cells utilize both enzymatic and nonenzymatic antioxidant mechanisms to diminish ROS levels and prevent oxidative damage. Superoxide dismutase (SOD), peroxidase (POD), ascorbate peroxidase (APX), catalase (CAT), and glutathione peroxidase (GPX) are antioxidant enzymes that work as O^2−^ and H_2_O_2_ scavengers [13, 14]. Nonenzymatic antioxidants, such as ascorbate, glutathione, phenols, and flavonoids, also play vital roles in ROS scavenging [15, 16].

Flavonoids are naturally occurring bioactive substances found in fruits, vegetables, tea, and medicinal plants [17]. Flavonoids comprise more than 9000 compounds and constitute a substantial category of plant secondary metabolites [18]. They have diverse biological functions in the growth and development of plants, including improving pollen fertility, imparting color, and influencing seed dormancy and germination [19, 20]. In addition, flavonoids have protective roles against biotic and abiotic stresses, such as pathogen infections, ultraviolet (UV)-B, cold, drought, and salinity [21–23]. Flavonoids have also received widespread attention due to their possible benefits for human health [24].

The molecular mechanism of flavonoid biosynthesis has been elucidated in many plants [25]. Chalcone synthase (CHS) mediates the first step in flavonoid production, catalyzing the formation of naringenin chalcone from three molecules of malonyl CoA and one molecule of 4-coumaroyl CoA. Chalcone isomerase (CHI) then quickly converts naringenin chalcone into naringenin (flavanone), which is further biosynthesized into different flavonoids by the subsequent enzymes in this pathway [26]. Although the biosynthesis of flavonoids has attracted increasing attention from scholars, current research does not fully explain the effects of regulatory factors on the transcription and activity of the major enzymes in flavonoid metabolism. Therefore, further research on the signaling molecules and regulatory pathways associated with flavonoids, as well as their regulatory mechanisms, is needed to elucidate the physiological activity of flavonoids.

*Rosa hybrida* cv. Jardin de Granville (JDG) is a new hybrid rose developed by 'Les Roses Anciennes André Eve' for the Prestige range of Christian Dior skin care products. JDG possesses twice the vitality of a traditional rose and grows and blooms vigorously in the salty air and harsh winds of coastal climates. JDG is also rich in beneficial bioactive substances that are mainly used in cosmetics and anti-aging skin care creams [27, 28]. *Rosa damascena* Mill. (DMS) is one of the most common fragrant roses in the Rosaceae family. Its essential oils and aromatic compounds are used extensively in the cosmetic and food industries worldwide [29]. DMS is considered an excellent rose throughout the world due to its high resistance to abiotic stress and abundance of beneficial secondary metabolites [30].

Here, we conducted an integrated analysis on the transcriptomes, proteomes, and metabolomes of JDG and DMS to explore the relationship between plant development and secondary metabolites of rose under salt stress. We used WGCNA and Cytoscape software to decipher the similarities and differences in the complex metabolic pathways and regulatory genes of JDG and DMS under salt stress. These results provide comprehensive information on the metabolic and molecular mechanisms of the response to salt stress in rose, promoting the cultivation of excellent new rose varieties that are both salt tolerant and rich in beneficial secondary metabolites.

## Results

### JDG is more tolerant than DMS to salt stress

**Figure 1:**
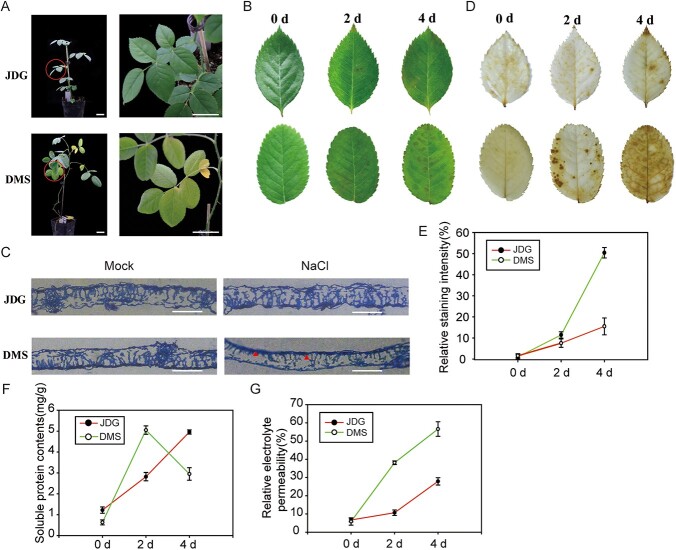
Phenotypes of JDG and DMS under salt stress. (A) Phenotypes of JDG and DMS plants after 2 weeks of treatment with 400 mM NaCl. Left, phenotype of the whole plant; right, enlarged image of the protruding part indicated by the red circle. Bars, 3 cm. (B) Detached leaves of rose on different days after onset of salt stress (400 mM NaCl). (C) Anatomical analysis of leaves in (B). Red arrowheads represent the palisade tissue. Mock (0 mM NaCl); NaCl (400 mM NaCl). Bars, 50 μm. (D) Tissue staining of rose leaves under salt stress using DAB. (E) Quantitative statistics of the relative staining intensity in (D). Brown staining area and total leaf area were measured using ImageJ software, their ratio is the relative staining intensity. (F) Soluble protein content of rose leaves at different days under salt treatment. (G) Relative electrolyte permeability of rose leaves at different days under salt treatment. Data are based on the mean ± SE of at least three repeated biological experiments.

To explore the salt tolerance of rose, plants of JDG and DMS were treated with 400 mM NaCl for 2 weeks. DMS plants showed typical damage with yellowing and death of leaves, while JDG leaves only exhibited slight wilting (Fig. 1A). Additionally, detached rose leaves were treated with salt for 4 days; DMS leaves showed significantly more necrosis than JDG leaves (Fig. 1B). In order to quickly observe the response of rose cultivars to salt stress and convenience sampling, subsequent experiments mainly used detached rose leaves. To examine the overall anatomy and morphology of leaves treated for 2 days with NaCl, we stained treated and control leaves with toluidine blue and prepared thin sections. Palisade tissue damage in response to salt treatment was more severe in DMS than in JDG (indicated by red arrowheads in Fig. 1C). To investigate ROS accumulation in response to salt stress, we performed 3, 3'-diaminobenzidine (DAB) staining. DMS leaves accumulated substantially more ROS (deeper staining) than JDG plants after salt stress, whereas there was no difference in ROS content between these two cultivars under normal conditions (Fig. 1D, E). Soluble protein content was higher in JDG leaves after 4 days of salt stress than after 2 days of salt stress, while the soluble protein content of DMS leaves was much higher than that of before treatment leaves after 2 days and decreased by 4 days of salt treatment (Fig. 1F). The relative electrolyte permeability of JDG leaves was increased slightly after 2 days of salt treatment and more substantially after 4 days of treatment, while relative electrolyte permeability was much higher in DMS than in JDG on both days after salt treatment (Fig. 1G). Phenotypic and physiological analyses indicated that JDG is more salt tolerant than DMS.

### Flavonoid metabolites play an important role in the salinity tolerance of rose

To better understand how salt stress affects rose metabolites, we performed a comprehensive untargeted analysis of metabolites using ultra-performance liquid chromatography/mass spectrometry (UPLC/MS). Fig. S1A shows the different metabolites detected, and Fig. S1B shows the curves of the quality control samples, indicating that the mass spectral data were highly reproducible and reliable. Principal component analysis (PCA) was used to reduce the data dimensions and clarify the relationships among the samples. The two principal components PC1, and PC2 could explain 50.07% and 23.36% of the variance, respectively. Moreover, PC1 revealed variance in genotypes, while PC2 revealed differences in time of exposure to salt stress. Thus, the metabolite-based PCA revealed obvious differences in salt tolerance between the two cultivars (Fig. S2A).

**Figure 2:**
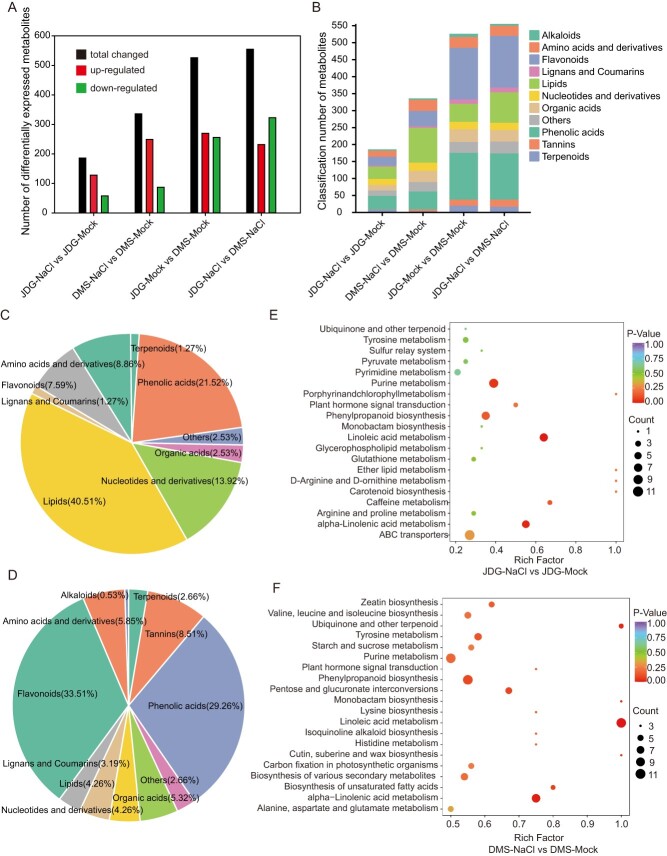
Metabolomic analysis of JDG and DMS under salt stress. (A) Number of DAMs in different comparison groups. (B) Classification of DAMs in each comparison. (C) Classification of DAMs upregulated in both JDG and DMS under salt treatment. (D) Classification of DAMs upregulated in JDG compared with DMS under both control and salt treatments. (E, F) KEGG pathway enrichment of DAMs under salt stress: (E) JDG-NaCl vs JDG-Mock and (F) DMS-NaCl vs DMS-Mock.

Our screening for differentially accumulated metabolites (DAMs) identified hundreds of metabolites with significantly altered accumulation under salt stress (Fig. 2A, Table S1). Preliminary analysis indicated that DAMs included amino acids and their derivatives, nucleotides and their derivatives, phenolic acids, flavonoids, lipids, tannins, lignans and coumarins, organic acids, alkaloids, and terpenoids, and most of the DAMs were upregulated under salt stress (Fig. 2B). Phenolic acids, lipids, and flavonoid metabolites showed significantly altered accumulation under salt stress in both JDG and DMS. Compared with their levels in DMS, flavonoid metabolites, phenolic acid metabolites, and lipids were differentially accumulated in JDG leaves under both control conditions and salt stress (Table S1). These results indicate that flavonoid metabolites, phenolic acid metabolites, and lipids may play important roles in the salt tolerance of rose.

To determine how metabolites differ between JDG and DMS, we summarized the differences in metabolite accumulation in the different comparison groups using Venn diagrams. Groups JDG-NaCl vs JDG-Mock and DMS-NaCl vs DMS-Mock shared 109 of the same metabolite changes, of which 79 were increases and 15 were decreases. Among the upregulated metabolites, phenolic acids and flavonoids accounted for 21.52% and 7.59%, respectively. These metabolites included ferulic acid, coniferaldehyde, pinocembrin (dihydrochrysin), naringin, eucalyptin (5-hydroxy-7,4'-dimethoxy-6,8-dimethylflavone), patuletin (quercetagetin-6-methyl ether), naringenin-7-*O*-rutinoside-4'-*O*-glucoside, naringin (naringenin-7-*O*-neohesperidoside), and sudachitin (Fig. 2C, Fig. S2B–D, Table S1). Notably, 5,7,8,4'-tetramethoxyflavone, vanillic acid-4-*O*-glucoside, and 3',4',5',5,7-pentamethoxyflavone were upregulated in JDG and downregulated in DMS under salt stress, while kaempferol-3-*O*-arabinoside-7-*O*-rhamnoside was upregulated in DMS and downregulated in JDG. Groups JDG-Mock vs DMS-Mock and JDG-NaCl vs DMS-NaCl shared 408 metabolites showing the same tendency in alteration, of which accumulation of 188 was increased and 202 was decreased. Among the upregulated metabolites, phenolic acids and flavonoids accounted for 29.26% and 33.51%, respectively (Fig. 2D). Notably, the genkwanin (apigenin 7-methyl ether) content was 12.74-fold higher, the 5,7-dihydroxy-6,3′,4′,5′-tetramethoxyflavone (arteanoflavone) content was 15.64-fold higher, the naringenin-4′,7-dimethyl ether content was 13-fold higher, and the naringin dihydrochalcone content was 13.30-fold in JDG compared with DMS under control conditions; all of these are flavonoid metabolites. Venn analysis also showed that many metabolites displaying changes under salt stress were genotype specific, indicating that the cultivars have different mechanisms of response to salinity. There were 77 metabolites that specifically accumulated in JDG under salt stress, which may represent the major metabolites in the salt stress response of JDG. Notably, four metabolites—ethylsalicylate (a phenolic acid), salidroside (a phenolic acid), L-ornithine (amino acids and derivatives), and epiafzelechin (a flavonoid)—accumulated specifically in JDG after salt treatment and were also highly accumulated under control conditions in JDG compared with DMS (Fig. S2B–D, Table S1).

All DAMs were analyzed using Kyoto Encyclopedia of Genes and Genomes (KEGG) pathway enrichment (Fig. 2E, F, Fig. S3A, B). In JDG (JDG-NaCl vs JDG-Mock group), salt stress induced changes in metabolites mainly involved 'purine metabolism,' 'phenylpropanoid biosynthesis,' 'linoleic acid metabolism,' and 'alpha-linolenic acid metabolism' (Fig. 2E). In DMS (DMS-NaCl vs DMS-Mock group), the DAMs in leaves under salt stress were mainly associated with 'phenylpropanoid biosynthesis,' 'alpha-linolenic acid metabolism,' 'linoleic acid metabolism,' and 'pentose and glucuronate interconversions' (Fig. 2F). In the JDG-Mock vs DMS-Mock group, DAMs between leaves of DMS and JDG were mostly associated with 'flavonoid biosynthesis,' 'flavone and flavonol biosynthesis,' and 'phenylpropanoid biosynthesis' (Fig. S3A). Meanwhile, in the JDG-NaCl vs DMS-NaCl group, DAMs were largely involved in 'flavonoid biosynthesis,' 'flavone and flavonol biosynthesis,' and 'linoleic acid metabolism' (Fig. S3B). KEGG enrichment analysis showed that 'linolenic acid/α-linolenic acid metabolism' and 'phenylpropanoid biosynthesis' were significantly enriched under salt stress in both cultivars, indicating that these two pathways play important roles under salt stress in rose. Regardless of the presence of salt stress, DAMs between DMS and JDG were concentrated in the flavone, flavonoid, and flavonol biosynthetic pathways, indicating that differential accumulation of these metabolites may be the main reason for different salt sensitivities among rose cultivars. Notably, 'caffeine metabolism' was enriched in JDG, while 'starch and sucrose metabolism' was significantly increased in DMS.

### Salt stress causes dynamic changes in distinct sets of proteins

**Figure 3:**
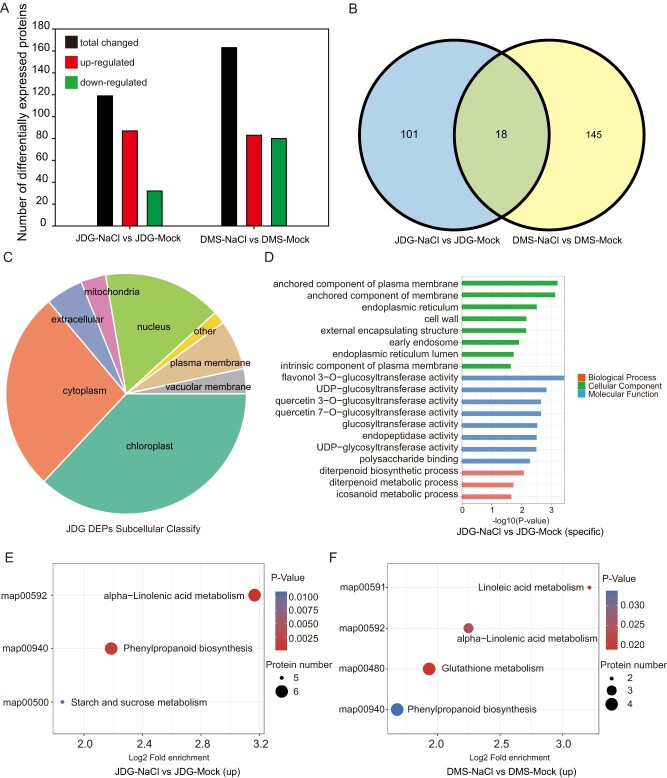
Proteomic analysis of rose under salt stress. (A) Number of DAPs in JDG and DMS. (B) Venn diagram of the DAPs in JDG and DMS. (C) Localizations of DAPs identified in JDG. (D) Functional categorization of DAPs unique to JDG. (E, F) KEGG enrichment analysis of DAPs in JDG (upregulated, E) and DMS (upregulated, F).

To delve deeper into the molecular mechanisms of the salt stress response in rose plants, we performed a proteome profiling analysis under the same salt treatment and control conditions as the metabolome analysis and characterized proteins on the basis of fold changes in their accumulation level. We identified 119 (87 upregulated and 32 downregulated) and 163 (83 downregulated and 80 upregulated) proteins with significantly differential accumulation under salt stress in JDG and DMS, respectively (Fig. 3A, B). Only 18 differentially accumulated proteins (DAPs) overlapped between the two cultivars, of which 13 were upregulated and 4 were downregulated in both JDG and DMS, while one DUF1279 domain–containing protein was upregulated in JDG and downregulated in DMS. Moreover, 101 DAPs were unique to JDG, whereas 145 DAPs were unique to DMS (Table S2).

We predicted that most of the DAPs are located in chloroplasts in rose, according to the WoLFPSORT database (Fig. 3C, Fig. S4A). Gene Ontology (GO) and KEGG analyses were performed to analyze and annotate protein functions. The 20 most highly enriched GO terms associated with the DAPs are depicted in a circle diagram (Fig. S5A, B, Table S2). Among them, GO:0046658 (anchored component of plasma membrane), GO:0051554 (flavonol metabolic process), GO:0047893 (flavonol 3-*O*-glucosyltransferase activity), and GO:0051555 (flavonol biosynthetic process) were highly enriched in JDG under salt stress. In DMS, GO:0006720 (isoprenoid catabolic process), GO:0005764 (lysosome), and GO:0004602 (glutathione peroxidase activity) were the most enriched among all GO terms. In addition, the GO data indicated that the DAPs specific to JDG were highly involved in the 'icosanoid metabolic process,' 'diterpenoid metabolic process,' and 'diterpenoid biosynthetic process' (Fig. 3D), whereas the DAPs specific to DMS were enriched in 'cellular hyperosmotic salinity response,' 'monocarboxylic acid catabolic process,' 'terpenoid catabolic process,' 'sesquiterpenoid catabolic process,' and 'apocarotenoid catabolic process' functions (Fig. S4B). DAPs shared by JDG and DMS included Q2VA35 (xyloglucan endotransglucosylase/hydrolase) and A0A2P6P708 (glutathione peroxidase), which are present only in extracellular regions (Table S2). The DAPs in different comparison groups were classified and then clustered according to enrichment of their associated GO terms (Fig. S4C). We determined that salinity mainly influences flavone and flavonol metabolism pathways in JDG. Flavones and flavonols are antioxidants and bioactive reagents [24]. In DMS, salt mainly influences the osmotic response, water stimulus response, and salt stress response pathways, most of which are stress related [31]. We used KEGG enrichment to determine the metabolic pathways associated with the DAPs in JDG and DMS under salt stress (Fig. 3E, F). Many DAPs in JDG were associated with phenylpropanoid biosynthesis and alpha-linolenic acid metabolism, with examples including lipoxygenase (A0A2P6S713), 12-oxophytodienoate reductase (A0A2P6PFD8), peroxidase (A0A2P6R8H8), and flavone 3′-*O*-methyltransferase (A0A2P6RK21). The DAPs upregulated in DMS under salt stress were frequently associated with alpha-linolenic acid metabolism and glutathione metabolism, whereas the DAPs that were downregulated were associated with ribosomes (Table S2). Notably, alpha-linolenic acid metabolism was significantly upregulated in both JDG and DMS under salt stress. Collectively, the GO and KEGG enrichment results show that salt stress causes dynamic changes in distinct sets of proteins in rose.

### Salt stress differentially alters the transcriptomes of JDG and DMS

**Figure 4:**
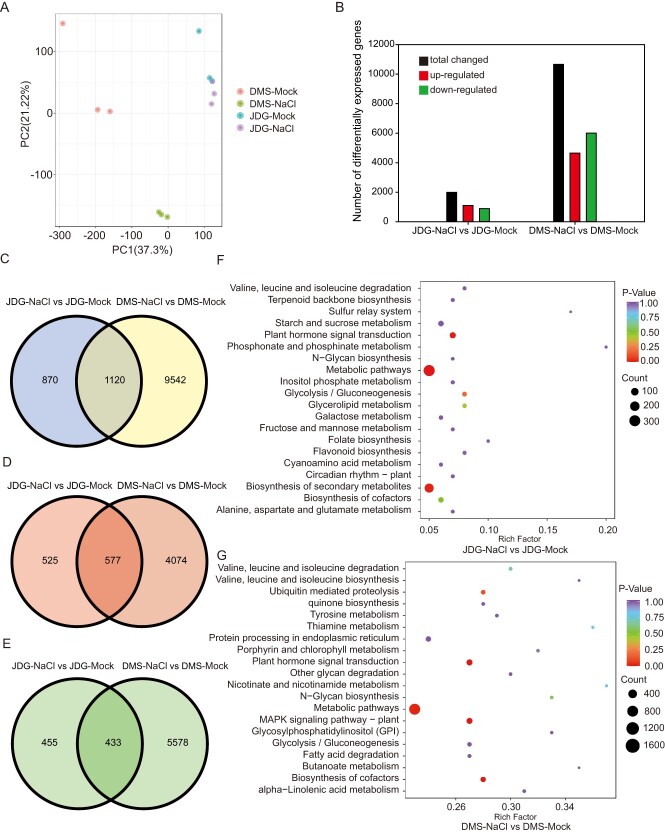
Transcriptomic analysis of JDG and DMS under salt stress. (A) PCA score plot of transcriptomic profiles from different cultivars. (B) Number of DEGs in JDG and DMS. (C–E) Venn diagrams of DEGs in JDG and DMS: (C) total DEGs, (D) upregulated DEGs, and (E) downregulated DEGs. (F, G) KEGG enrichment analysis of DEGs in JDG (F) and DMS (G).

To identify the genes involved in salt stress and explore the molecular mechanisms of salt tolerance in DMS and JDG, we sequenced the transcriptomes of JDG and DMS leaves by RNA sequencing (RNA-seq). We obtained high-quality reads for transcriptome analysis (Table S3). PCA showed a distinct difference between the two cultivars along PC1, and PC2 separated the treatment from the control. The three biological replicates in the ordination space were mostly clustered together, suggesting an acceptable correlation between replicates (Fig. 4A).

**Figure 5:**
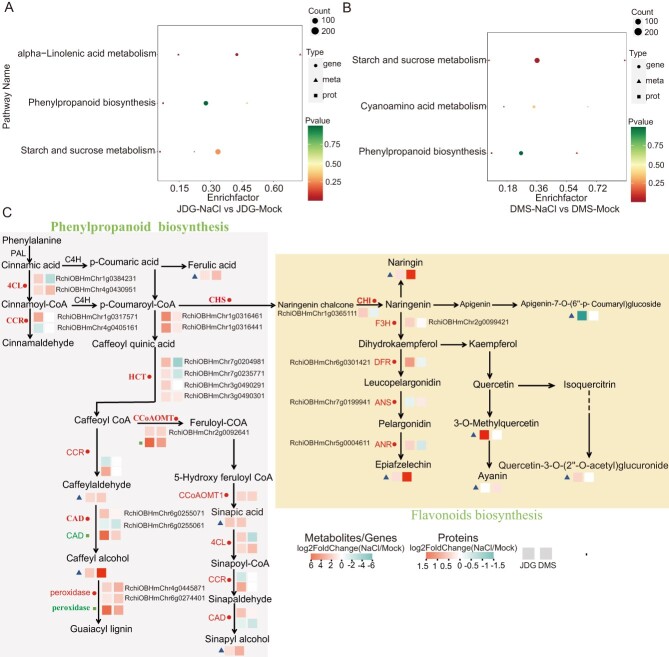
Correlation analysis of transcriptome, proteome, and metabolomics data. (A, B) KEGG enrichment analysis of combined transcriptome, proteome, and metabolome data: (A) JDG-NaCl vs JDG-Mock, and (B) DMS-NaCl vs DMS-Mock. The x-axis shows the enrichment factor of the pathway in different omics, and the y-axis shows the name of the KEGG pathway; the color from red to green represents the significance of enrichment from high to low (indicated by the *P* value). The size of bubbles indicates the number of DEGs, DAPs, or DAMs; the larger the number, the larger the symbol. The shape of bubbles illustrates the various omics: circles represent genes omics, triangles represent metabolites omics, and squares represent proteins omics. (C) Co-expression network of major genes, proteins, and metabolites in the phenylpropanoid pathway. Different colors indicate the value of log_2_Fold Change (NaCl/Mock), with red for upregulated and blue for downregulated genes, proteins, or metabolites.

We analyzed differentially expressed genes (DEGs) in JDG and DMS under control and salt stress conditions. We detected 10,662 DEGs in DMS under salt stress, of which 4651 were upregulated and 6011 were downregulated. However, only 1990 genes were differentially expressed in JDG: 1102 upregulated and 888 downregulated (Fig. 4B). The smaller number of DEGs in JDG than in DMS under salt stress implies that JDG is less affected by salt stress. We used a Venn diagram to display the differences between various genes in DMS and JDG under salt stress. Group DMS-NaCl vs DMS-Mock and group JDG-NaCl vs JDG-Mock shared 1120 DEGs under salt stress, with 577 upregulated genes and 433 downregulated genes (Fig. 4C–E).

Next, we performed GO analysis of DEGs in the categories cellular component (CC), biological process (BP), and molecular function (MF). The top 21 most enriched GO terms associated with DEGs of JDG-NaCl vs JDG-Mock and DMS-NaCl vs DMS-Mock are presented in circle diagrams (Fig. S6, Table S4). Seven GO terms associated with the JDG-NaCl vs JDG-Mock group were highly involved in the BP category, among which GO:0016052 (carbohydrate catabolic process), GO:0009813 (flavonoid biosynthetic process), and GO:0009812 (flavonoid metabolic process) contained the most DEGs (43, 26, and 27, respectively), and most of these enriched genes were upregulated. Thirteen GO terms were highly involved in the MF category, among which GO:0010427 (abscisic acid binding), GO:0016832 (aldehyde-lyase activity), and GO:0019840 (isoprenoid binding) were highly significant. One GO term was highly involved in the CC category: GO:0031226 (intrinsic component of plasma membrane). Moreover, 19 GO terms associated with the DMS-NaCl vs DMS-Mock group were enriched in the BP category, among which GO:0036294 (cellular response to decreased oxygen levels), GO:0048511 (rhythmic process), and GO:0048585 (negative regulation of response to stimulus) contained the most DEGs (85, 95, and 146, respectively), and most of these enriched genes were downregulated. One GO term was enriched in the MF category: GO:0016854 (racemase and epimerase activity). Similarly, one GO term was enriched in the CC category: GO:0009501 (amyloplast). KEGG pathway enrichment analysis for JDG-NaCl vs JDG-Mock revealed that the DEGs were mainly involved in metabolic pathways, plant hormone signal transduction, biosynthesis of secondary metabolites, and glycolysis/gluconeogenesis (Fig. 4F, Table S4). In the DMS-NaCl vs DMS-Mock group, the DEGs were chiefly enriched in metabolic pathways, plant hormone signal transduction, the MAPK signaling pathway, biosynthesis of cofactors, and ubiquitin-mediated proteolysis (Fig. 4G, Table S4). These findings indicate that the biosynthesis of secondary metabolites is substantially enhanced under salt stress in JDG, but not in DMS. However, the biosynthesis of cofactors associated with primary metabolism is enhanced under salt stress in DMS. Therefore, we speculate that salinity results in large changes in primary metabolism in DMS, while it influences secondary metabolism in JDG.

Transcription factors (TFs) are essential for regulating the expression of stress response genes. Among the DEGs, we identified 114 TFs in JDG and 491 TFs in DMS, covering 39 TF families (Table S4). The most abundant genes belonged to the AP2/ERF-ERF, MYB, NAC, bHLH, and C2C2 families (Fig. S7A, B). Moreover, 64 TFs were differentially expressed in both cultivars in response to salinity. We speculate that these TFs form a highly complex transcriptional regulatory network and could perform critical functions in the mechanism of salt tolerance in rose.

### Expression of phenylpropanoid-related genes is correlated with proteins and metabolites affected by salt stress

Integrated analysis of multi-omics data provides a powerful tool for identifying significantly different pathways and crucial metabolites in biological processes. Here, we integrated our transcriptome, proteome, and metabolome data to determine the performance of the two rose cultivars under salt stress. Pathways associated with alpha-linolenic acid metabolism, phenylpropanoid biosynthesis, and starch and sucrose metabolism were significantly enriched in JDG under salt stress (Fig. 5A), while the pathways enriched in DMS were involved in starch and sucrose metabolism, cyanoamino acid metabolism, and phenylpropanoid biosynthesis (Fig. 5B). Starch and sucrose metabolism represent primary metabolic functions common to different cultivars [32], while alpha-linolenic acid metabolism is related to the biosynthesis of jasmonic acid, which is a phytohormone involved in fungal invasion and senescence [7]. The phenylpropanoid biosynthesis pathway comprises multiple secondary metabolites, which confer a range of colors, flavors, nutritional components, and bioactivities in plants. Flavonoids are an important type of phenylpropanoid that play key roles in resistance against biotic and abiotic stresses [24]. Thus, we focused on the phenylpropanoid pathway.

Gene–protein–metabolite correlation networks can be used to elucidate functional relationships and identify regulatory factors. Therefore, we analyzed the regulatory networks of the DEGs, DAPs, and DAMs related to phenylpropanoid metabolism. We identified 14 DEGs that were strongly correlated with one DAP and six DAMs in JDG under salt stress. Similarly, 25 DEGs were strongly correlated with one DAP and eight DAMs in DMS under salt stress (Table S5). For example, in JDG, there was a strong correlation between the expression of one gene (RchiOBHmChr4g0430951) and the abundance of one protein (A0A2P6PM56) and two metabolites [coniferyl alcohol (mws0093) and sinapyl alcohol (mws0853)]. Epiafzelechin (mws1422) was also significantly associated with the expression of the gene RchiOBHmChr2g0092641. In DMS, there was a close association between the expression of three genes (RchiOBHmChr2g0092671, RchiOBHmChr3g0480401, and RchiOBHmChr5g0041231) and the abundance of one protein (A0A2P6QM41) and one metabolite [L-tyrosine (mws0250)]. The strong association of particular genes with phenylpropanoid proteins or metabolites suggests that these genes play a major role in phenylpropanoid biosynthesis under salt stress.

We selected 20 important genes in the biosynthetic pathway of phenylpropanoid and compared their expression between rose cultivars (Table S6). The transcript levels of many genes (*4CL1*, *CCR1*, *HCT1*, *HCT2*, *HCT3*, *HCT4*, *CHS1*, *CHS2*, *CHI*, *DFR*, *F3H*, and *ANR*) were higher in JDG than in DMS, which may be valuable for salt tolerance by stimulating JDG to produce more flavonoids. Our multi-omics analysis revealed that ferulic acid, sinapic acid, and coniferaldehyde accumulated to high levels in JDG under salt stress (Fig. 5C, Table S1). We also compared the flavonoid compounds in the two cultivars. Quercetin-3,3′-dimethyl ether, 5,7-dihydroxy-6,3′,4′,5′-tetramethoxyflavone (arteanoflavone), naringenin-4′,7-dimethyl ether, naringin dihydrochalcone, genkwanin (apigenin 7-methyl ether), and mearnsetin accumulated to greater levels in JDG than in DMS under control conditions. Correspondingly, the flavonoids brickellin, 3-*O*-methylquercetin, 5,2′,5′-trihydroxy-3,7,4′-trimethoxyflavone-2′-*O*-glucoside, and kaempferol-3-*O*-(6′′-acetyl)glucosyl-(1→3)-galactoside were more abundant in JDG than in DMS under salt stress. By contrast, naringenin-4′,7-dimethyl ether, aromadendrin (dihydrokaempferol), pinocembrin-7-*O*-(6′′-*O*-malonyl)glucoside, Quercetin-3-*O*-(2”-*O*-glucosyl)glucuronide, were specifically accumulated in DMS. Moreover, 3′,4′,5′,5,7-pentamethoxyflavone, 3,5,7,3′4′-pentamethoxyflavone, and 5,7,8,4′-tetramethoxyflavone were abundant in JDG under salt stress but were decreased in DMS (Table S7). Overall, the integration of the three omics datasets indicated that the phenylpropane pathway, especially the flavonoid pathway, is strongly enhanced under salinity conditions and that this contributes to salt tolerance in roses, especially in the JDG genotype.

### Networks of co-expressed genes associated with phenylpropanoid biosynthesis are involved in the salt stress response

**Figure 6:**
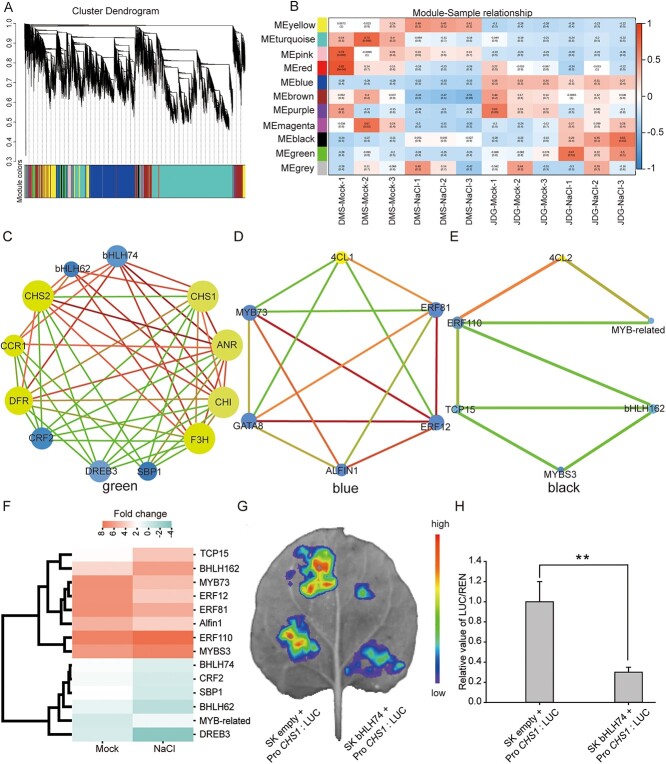
Co-expression network related to flavonoid biosynthesis. **(A)** Clustering tree based on the correlation between gene expression levels. (B) Module–sample relationships. Each row represents a gene module, with the same color in as (A); each column represents a sample; the boxes within the chart contain corresponding correlations and *P* values. (C–E) Networks built from correlations among structural genes and TFs. Circles represent genes, and the size of the circle represents the number of relationships between genes in the network and surrounding genes. Lines represent regulatory relationships between genes, and different colored lines represent different connection strengths: red, strong connections; green, weak connections. (F) Heat map depicting the expression profiles of 15 TF genes. The scale bar denotes the Fold change/(mean expression levels across the three treatment groups). The color indicates relative levels of gene expression, horizontal rows represent the different treatments in JDG, and vertical columns show the TFs. (G) Representative images of transient expression of *bHLH74* and *LUC* driven by the *CHS1* promoter in *Nicotiana benthamiana* leaves. The color scale represents the signal level. High represents a strong signal, and low represents a weak signal. (H) Relative value of LUC/REN. Data are based on the mean ± SE of at least three repeated biological experiments. Significance determined using Student’s *t*-test (^**^P < 0.01).

To identify candidate genes associated with phenylpropanoid biosynthesis, we constructed co-expression gene network modules via weighted gene correlation network analysis (WGCNA). We constructed a cluster tree based on correlation between expression levels (indicated by fragments per kilobase of script per million fragments mapped, FPKM), which partitioned the genes into 11 different gene modules (Fig. 6A, B). To identify candidate genes that play significant roles within the gene networks, we extracted annotation information for all these genes from the *Rosa chinensis* 'Old Blush' reference genome annotation database. We selected 16 genes contributing to phenylpropanoid biosynthesis and four genes associated with flavonoid biosynthesis. Table S8 lists the annotated genes participating in flavonoid-related pathways in JDG. Among the 11 modules, the green module contained 10 of these genes: *CHS1*, *CHS2*, *CCR1*, *HCT3*, *HCT4*, *CCoAOMT*, *F3H*, *DFR*, *ANR*, and *CHI*. The turquoise module contained three genes: *CCR2*, *HCT1*, and *CAD2*. The blue module contained three genes: *PRDX1*, *4CL1*, and *ANS*. The red, yellow, brown, and black modules each contained one gene: *CAD1*, *PRDX2*, *HCT2*, and *4CL2*, respectively (Table S8). After combining certain genes in modules and comparing them with the DEGs, we checked and confirmed these results using reverse-transcription quantitative PCR (RT-qPCR). The expression trends of eight DEGs from phenylpropanoid and flavonoid biosynthesis pathways matched the results of RNA-seq (Fig. S8).

To determine the regulatory genes involved in phenylpropanoid biosynthesis in JDG, we constructed three subnetworks from the different modules using the 20 phenylpropanoid biosynthesis–related DEGs as the nodes (Table S9). In the regulatory networks of phenylpropanoid biosynthesis, we identified 15 TF genes from seven TF families: AP2/ERF-ERF (5 unigenes), bHLH (3 unigenes), MYB (3 unigenes), Alfin-like (1 unigene), SBP (1 unigene), C2C2-GATA (1 unigene), and TCP (1 unigene). *bHLH62* and *bHLH74* were strongly associated with *CHS1*, *CHS2*, *CHI*, *CCR1*, and *F3H*; *ERF81* was strongly associated with *4CL1*; and *ERF110* and *MYB-related* were strongly associated with *4CL2* (Fig. 6C–E), indicating that *CHS* and *4CL* are the major target genes in phenylpropanoid biosynthesis. Therefore, we speculated that the abundance of flavonoids is increased by enhancing the expression of upstream flavonoid biosynthesis genes. Fig. 6F shows a heat map of expression of the 15 TF genes after NaCl treatment. The green module contained a substantial number of phenylpropanoid biosynthesis genes, among which *CHS1* was closely related to the TFs bHLH74 and bHLH62. Therefore, dual-luciferase reporter assays were conducted to determine their regulatory relationship (Fig. 6G, H). We used *bHLH74* and *bHLH62* driven by the CaMV35S promoter as effectors in a transient expression system, with the *CHS1* promoter fused with *LUC* as a reporter. When we cotransformed *Nicotiana benthamiana* leaves with the effectors and the reporter, the LUC/REN ratio of CHS1 was 0.3/1, which was drastically lower than those of the controls (Fig. 6G, H, Fig. S9A, B). These results indicate that bHLH74, but not bHLH62, inhibits the expression of *CHS1*.

## Discussion

### Salt stress damages the structure and osmotic potential of rose leaves

Roses belong to the Rosaceae family and are one of the most important commercial flower crops. Extracts from various parts of the rose plant have also been shown to have excellent biological activity and are used in industries such as cosmetics, perfume and medicine [1]. Meanwhile, an increasing number of wild rose varieties with significant health benefits are being domesticated and brought into mainstream cultivation [33]. Salt stress is one of the most widespread abiotic constraints for rose cultivation. Salt stress threatens plant survival and growth but can stimulate an increase in the biosynthesis of secondary metabolites [34]. Previous studies have shown that optimal coordination between leaf structure and photosynthetic processes is essential for enabling plants to tolerate salt stress [35]. When exposed to salt treatment, leaves become thicker and smaller while the palisade tissue and spongy tissue become loose and jumbled and the intercellular space of the mesophyll becomes thinner [36–39]. We observed that the palisade tissue of DMS was loose, disordered, and severely damaged compared with that in JDG under salt stress (Fig. 1C). This indicates that DMS is more sensitive to salt stress than JDG. Typically, excessive ROS accumulate under stress conditions, which can lead to membrane oxidative damage (lipid peroxidation) [40]. Silencing of the gene *GmNAC06* in soybean (*Glycine max*) leads to accumulation of ROS under salt stress, which in turn leads to significant losses in soybean production [41]. In *Arabidopsis*, the *sibp1* mutant accumulates more ROS than wild-type plants or AtSIBP1-overexpressing plants, resulting in a lower survival rate under salt treatment [42]. In this study, salinity led to a greater accumulation of ROS in DMS compared with JDG, as detected by DAB staining (Fig. 1D, E). This indicates that DMS suffers greater damage under salinity stress. Excessive accumulation of ROS in cells can lead to membrane oxidative damage and trigger the production of enzyme systems or non-enzyme free radical scavengers to cope with oxidative damage [10]. Here, antioxidant enzyme activities such as peroxidase (A0A2P6R8H8) and glutathione peroxidase (A0A2P6P708) were upregulated in roses under salt treatment (Table S2). This suggests that rose plants maintain lower ROS levels by upregulating the activity of antioxidant enzymes, thereby protecting photosynthetic mechanisms and maintaining plant growth under salt stress. Among the nonenzymatic antioxidants, phenols and flavonoids accumulate in various tissues and contribute to free radical scavenging that enhances plant salt tolerance [43]. Indeed, we identified significant differences in the contents of phenolic acids, lipids, and flavonoid metabolites in JDG and DMS under control and salt stress conditions (Table S1). Moreover, our transcriptomic and proteomic analysis revealed the activation of genes and proteins within the phenylpropanoid and flavonol pathways. This activation results in the accumulation of various phenolic compounds, potentially enhancing their capacity for scavenging ROS.

### Flavonoids are beneficial for improving salt stress in rose

Phenolic compounds, such as flavonoids, are among the most widespread secondary metabolites observed throughout the plant kingdom [44]. These compounds fulfill various biochemical and molecular functions within plants, encompassing roles in plant defense, signal transduction, antioxidant action, and the scavenging of free radicals [45]. Environmental changes commonly trigger the flavonoid pathway, which aids in shielding plants from the harmful effects of ultraviolet radiation, salt, heat, and drought [23, 46, 47]. Moreover, flavonoids demonstrate potent biological activity and serve as significant antioxidants [48]. Recently, researchers and consumers have been interested in plant-based polyphenols and flavonoids for their antioxidant potential, their dietary accessibility, and their role in preventing fatal diseases such as cardiovascular disease and cancer [49]. Our transcriptomics analysis showed that salinity causes significant alterations in the secondary metabolism of JDG, while affecting the primary metabolism of DMS. Proteomics showed that phenylpropanoid biosynthesis is significantly enhanced in JDG under salt stress, especially through the flavonoid pathway. In DMS, glutathione metabolism is significantly enhanced under salt stress, indicating differences in salt tolerance pathways between the two cultivars. Our metabolome data indicated that the abundance of phenolic acid and flavonoid metabolites was significantly altered in both JDG and DMS under salt stress. Furthermore, by comparing their contents in leaves under salt stress and control conditions, we found that more flavonoids accumulated in DMS than in JDG under salt stress. This evidence suggests that DMS requires an increased presence of flavones to withstand the damage caused by salinity. By contrast, salinity stress did not trigger a substantial buildup of flavonoids in JDG, possibly due to the adequate levels of flavonoids already present under normal conditions, which provided ample tolerance to salt-induced stress. This observation could also explain the higher tolerance of JDG to salt stress (Table S1). When we compared the flavonoid metabolites of the phenylpropanoid pathway to identify flavonoid metabolites associated with salt tolerance, we found that 17 phenolic acid metabolites and 6 flavonoid metabolites were significantly differentially accumulated in both genotypes. Of these compounds, ferulic acid serves as a free radical scavenger, while simultaneously serving as an inhibitor for enzymes engaged in generating free radicals and boosting the activity of scavenger enzymes [49]. Sinapic acid is a bioactive phenolic acid with anti-inflammatory and anti-anxiety effects [50]. Pinocembrin, a naturally occurring flavonoid found in fruits, vegetables, nuts, seeds, flowers, and tea, is an anti-inflammatory, antimicrobial, and antioxidant agent [51]. This indicates that these two rose cultivars contain beneficial metabolites with some economic value. We investigated the possible effects of these metabolites in conferring salt tolerance in rose by comparing specific DAMs between JDG and DMS. Among these DAMs, eight metabolites were upregulated and six metabolites were downregulated under salt treatment in JDG compared to DMS. Among these eight upregulated DAMs, the contents of 3-*O*-methylquercetin, brickellin, 5,2′,5′-trihydroxy-3,7,4′-trimethoxyflavone-2′-*O*-glucoside, and kaempferol-3-*O*-(6′′-acetyl)glucosyl-(1→3)-galactoside accumulated significantly with salinity (Table S7). These metabolites have important functions. For example, 3-*O*-methylquercetin has potent anticancer, antioxidant, antiallergy, and antimicrobial activities and shows strong antiviral activity against tomato ringspot virus [52]. Kaempferol, a biologically active compound found in numerous fruits, vegetables, and herbs, demonstrates various pharmacological benefits, such as antimicrobial, antioxidant, and anticancer properties [53]. This indicates that JDG is an excellent rose cultivar that is both salt tolerant and rich in beneficial bioactive substances.

### bHLHL74 regulates flavonoid biosynthesis

The biosynthesis of flavonoids is initiated from the amino acid phenylalanine, giving rise to phenylpropanoids that subsequently enter the flavonoid-anthocyanin pathway [25]. The CHS enzyme is situated at a crucial regulatory position preceding the flavonoid biosynthetic pathway, directing the flow of the phenylpropanoid pathway towards flavonoid production, which has been extensively documented in many plant species [54, 55]. In rice (*Oryza sativa*), defects in the flavonoid biosynthesis gene *CHS* can alter the distribution of flavonoids and lignin [56]. In eggplant (*Solanum melongena* L.), *CHS* regulates the content of anthocyanins in eggplant skin under heat stress [57]. In apple (*Malus domestica*), overexpression of *CHS* increases the accumulation of flavonoids and enhances nitrogen absorption [58]. We identified a positive correlation between flavonoid accumulation and the expression of *CHS* genes, in agreement with previous reports. The bHLH TFs involved in regulating flavonoid biosynthesis work in a MYB-dependent or -independent manner. For example, DvIVS, a bHLH transcription factor in dahlia (*Dahlia variabilis*), activates flavonoid biosynthesis by regulating the expression of *Chalcone synthase 1* (*CHS1*) [59]. The *Arabidopsis* bHLH proteins TRANSPARENT TESTA 8 (AtTT8) and ENHANCER OF GLABRA 3 (AtEGL3) are all involved in the biosynthesis of various flavonoids [60–62]. In Chrysanthemum (*Chrysanthemum morifolium*), CmbHLH2 significantly activates *CmDFR* transcription, leading to anthocyanin accumulation, especially when in coordination with CmMYB6 [63]. In blueberry (*Vaccinium* sect. *Cyanococcus*), the bHLH25 and bHLH74 TFs potentially engage with MYB or directly hinder the expression of genes responsible for flavonoid biosynthesis, thereby regulating flavonoid accumulation [64]. In apple (*Malus domestica*), expression of bHLH62, bHLH74, and bHLH162 is significantly negatively correlated with anthocyanin content and has been shown to inhibit anthocyanin biosynthesis [65]. In apple fruit skin, hypermethylation of bHLH74 in the mCG context leads to transcriptional inhibition of downstream anthocyanin biosynthesis genes [66]. In rose, our co-expression network revealed a strong correlation between *CHS* and genes encoding TFs such as *bHLH74* and *bHLH62* in the key gene network. bHLH proteins can bind to the promoter regions of pivotal genes encoding enzymes, playing important roles in regulating DAMs under salt stress. Dual-luciferase reporter assays showed that LUC bioluminescence was suppressed well below background levels in *Nicotiana benthamiana* leaves infiltrated with pCHS1:LUC plus 35S:bHLH74, but not 35S:bHLH62 (Fig. 6G, H, Fig. S9A, B). Thus, we conclude that bHLHL74 TFs negatively regulate flavonoid biosynthesis by directly inhibiting the expression of *CHS1*, which is involved in the flavonoid biosynthetic pathway.

## Conclusions

We examined the morphological phenotypes, transcriptomes, proteomes, and widely targeted metabolomes of JDG and DMS under salt stress. Multi-omics analysis revealed that the phenylpropane pathway, especially the flavonoid pathway, contributes strongly to salt tolerance in rose, particularly JDG. Meanwhile, the bHLHL74 TF negatively regulates flavonoid biosynthesis by repressing the expression of the *CHS1* gene involved in the flavonoid biosynthetic pathway. This research facilitates our understanding of the regulatory mechanisms of plant development and secondary metabolites underlying salt stress responses in rose, offering valuable insights that could be used to develop new strategies for improving plant tolerance to salinity.

## Materials and methods

### Plant materials and growth conditions

*Rosa hybrida* cv. Jardin de Granville (JDG) and *Rosa damascena* Mill. (DMS) were planted in the Science and Technology Park of China Agricultural University (40°03′N, 116°29′E). Rose plants were propagated by cutting culture. Rose shoots with at least two nodes and approximately 6 cm in length were used as cuttings and inserted into square flowerpots (diameter 8 cm) containing a mixture of vermiculite and peat soil [1:1 (v/v)]. Cuttings were soaked in 0.15% (v/v) indole-3-butytric acid (IBA) before insertion into pots and then grown in a growth chamber at 25°C with 50% relative humidity and a cycle of 8 hours of darkness/16 hours of light for 1 month until rooting [67].

*Nicotiana benthamiana* plants were used for measurement of transient expression. Seeds were sown in square flowerpots (diameter 8 cm); after 1 week, seedlings were transplanted into different pots. The soil and cultivation conditions for *N. benthamiana* cultivation were the same as those for roses.

### Salt treatment

Twenty JDG and 20 DMS rose cuttings displaying good rooting and uniform appearance were selected for salt treatment experiments. JDG or DMS plants were randomly divided into two groups watered with either 0 or 400 mM NaCl. Phenotypes were recorded after 2 weeks. This process was repeated three times [68].

Salt treatment of rose leaves was described previously [68]. Thirty JDG and 30 DMS rose cuttings with good rooting and uniform appearance were selected, and mature leaves of similar size were collected. The leaves were divided into two treatment groups, each containing 30 leaves: group A, immersed in deionized water treatment, and group B, immersed in 400 mM NaCl treatment. Phenotypes were observed after 0, 2, and 4 days. On the second day of treatment, leaves showed obvious differences. By the fourth day of treatment, the leaves had become soft or had died. Therefore, sequencing data from the second day were used. Three independent biological replicates were assayed.

### Relative electrolyte permeability

Determination of relative electrolyte permeability was as previously reported [69] with the following modifications. Salt-treated leaves (0.1 g) were weighed, placed in a 50-ml centrifuge tube, and covered with 20 ml deionized water. The conductivity of the distilled water was measured and defined as EC0. After shaking for 20 minutes at 60 rpm on an orbital shaker, the conductivity at room temperature was measured and defined as EC1. The centrifuge tube was then placed in boiling water for 10 minutes and cooled to room temperature, and the conductivity of the solution was measured as EC2. The relative permeability of the electrolytes (as a percentage) was determined as (EC1-EC0) / (EC2-EC0) × 100%.

### Soluble protein content

Soluble protein content was determined following the method of Bradford (1976) [70]. Leaf samples (0.5 g) were placed in a mortar with 8 ml distilled water and a small amount of quartz sand, crushed thoroughly, and incubated at room temperature for 0.5 hours. After centrifugation at 3,000 *g* for 20 minutes at 4 °C, the supernatant was transferred to a 10-ml volumetric flask and the volume was adjusted to 10 ml with distilled water. Two 1.0-ml aliquots of this sample extraction solution (or distilled water as a control) were transferred to clean test tubes, 5 ml of Coomassie Brilliant Blue reagent was added, and the tubes were shaken well. After 2 minutes, when the reaction was complete, the absorbance and chromaticity at 595 nm were measured, and the protein content was determined using a standard curve.

### Leaf anatomical structure

Paraffin sections were prepared as described previously with some modifications [71]. Leaves from the control and NaCl treatments were collected, washed slowly with deionized water at normal room temperature, and stored at 4°C until further use. A 3-mm × 5-mm sample was cut from the same part of each leaf, and these leaf samples were fixed in 2.5% (v/v) glutaraldehyde. Samples were dehydrated using acetone through a concentration gradient of 30%, 50%, 70%, 80%, 95%, and 100% (v/v) and then embedded in paraffin. The embedded tissues (3-μm sections) were sectioned using a Leica RM2265 rotary slicer (Leica Microsystems, Wetzlar, Germany). Slides were stained with 0.02% (v/v) toluidine blue for 5 minutes, and the residual toluidine blue was removed using distilled water. Slides were allowed to dry and then observed under a microscope (OLYMPUS BH-2, Tokyo, Japan). Three independent biological replicates were examined.

### DAB (3,3′-diaminobenzidine) staining for H_2_O_2_

H_2_O_2_ content was detected using the DAB staining method [72]. Leaves treated with NaCl or control leaves were rinsed clean with distilled water, immersed in DAB solution (1 mg/ml, pH 3.8), and placed under vacuum at approximately 0.8 Mpa for 5 minutes; this process was repeated three to six times until the leaves were completely infiltrated. Leaves were then incubated in a box in the dark for 8 hours until a brown sediment was observed. Chlorophyll was removed by repeatedly washing with eluent (ethanol:lactic acid:glycerol, 3:1:1, v/v/v). Decolorized leaves were photographed to record their phenotypes. ImageJ was used to quantify the stained areas.

### UPLC-QQQ-based widely targeted metabolome analysis

Metabolomics analysis was performed on four groups of samples: JDG-Mock, JDG-NaCl, DMS-Mock, and DMS-NaCl. Extraction and determination of metabolites were performed with the assistance of Wuhan Metware Biotechnology Co., Ltd. Samples were crushed using a stirrer containing zirconia beads (MM 400, Retsch). Freeze-dried samples (0.1 g) were incubated overnight with 1.2 ml 70% (v/v) methanol solution at 4 °C, then centrifuged at 13,400 *g* for 10 minutes. The extracts were filtered and subjected to LC-MS/MS analysis [73]. A previously described procedure [74] was followed for analyzing the conditions and quantifying metabolites using an LC-ESI-Q TRAP-MS/MS in multi-reaction monitoring (MRM) mode. The prcomp function was used for PCA, significantly different metabolites were determined by |log_2_Fold Change| ≥ 1, and annotated metabolites were mapped to the KEGG pathway database (http://www.kegg.jp/kegg/pathway.html). Comparisons are described as follows: e.g., JDG-NaCl vs JDG-Mock, indicating that the treated sample is being compared with the untreated sample and that metabolites are upregulated or downregulated in the NaCl sample compared with the Mock sample.

### Tandem mass tag-based proteomic analysis

Experiments were carried out with the assistance of Hangzhou Jingjie Biotechnology Co., Ltd. Samples were thoroughly ground into powder using liquid nitrogen, and protein extraction was performed using the phenol extraction method. The protein was added to trypsin for enzymolysis overnight, and then the peptide segments were labeled with TMT tags. LC-MS/MS analysis was performed using an EASY-nLC 1200 UPLC system (ThermoFisher Scientific) and a Q Active^TM^ HF-X (ThermoFisher Scientific) [75]. An absolute value of 1.3 was used as the threshold for significant changes. GO (http://www.ebi.ac.uk/GOA/) and KEGG categories were used to annotate DAPs; WoLFPSORT software was used to predict subcellular localization (https://wolfpsort.hgc.jp/).

### Transcriptome sequencing

We constructed 12 cDNA libraries (three biological replicates for each of JDG and DMS under each treatment) for RNA-seq. Transcriptome sequencing was completed at Wuhan Metware Biotechnology Co., Ltd. RNA purity and RNA integrity were determined using a nanophotometer spectrophotometer and an Agilent 2100 bioanalyzer, respectively. The RNA library was then sequenced on the Illumina Hiseq platform. Raw data were filtered using fastp v 0.19.3 and compared with the reference genome (https://lipm-browsers.toulouse.inra.fr/pub/RchiOBHm-V2/). FPKM (fragments per kilobase of script per million fragments mapped) was used as an indicator to measure gene expression levels, with the threshold for significant differential expression being an absolute |log_2_Fold Change| ≥ 1 and False Discovery Rate < 0.05. GO and KEGG categories were used to annotate DEGs [76].

To identify modules with high gene correlation, co-expression network analysis was performed using the R-based WGCNA package (v.1.69) with default parameters [77]. The varFilter function of the R language genefilter package was used to remove genes with low or stable expression levels in all samples. Modules based on the correlation between gene expression levels were identified, and a correlation matrix between each module and the sample was calculated using the R-based WGCNA software package. The module network was visualized using Cytoscape software (v.3.7.2).

### RT-qPCR

RT-qPCR was performed on eight DEGs in the phenylpropanoid pathway to verify the accuracy of the data obtained from high-throughput sequencing. Total RNA was extracted using the hot borate method [72] and reverse transcribed using HiScript III All-in-one RT SuperMix (R333-01, Vazyme Biotech Co., Ltd., Nanjing, China). Subsequently, 2 × ChamQ SYBR qPCR Master Mix (Q331, Vazyme Biotech Co., Ltd., Nanjing, China) was used for quantitative detection of gene expression. The relative expression of genes was calculated using the 2^−ΔΔCt^ method [76]. *GAPDH* was used as an endogenous control, and primers for RT-qPCR are listed in Table S10.

### Dual-LUC reporter assay

A transactivation assay was designed to evaluate the effect of BHLH74/BHLH62 on the *CHS1* promoter using methods described previously [78]. Initially, a 2000-bp segment of the *CHS1* promoter was cloned into the pGreenII 0800-LUC vector, generating the ProCHS1:LUC reporter plasmid. Concurrently, the coding sequences of BHLH74/BHLH62 were inserted into the pGreenII0029 62-SK vector, resulting in the construction of Pro35S: BHLH74/BHLH62 effector plasmids. pGreenII 0800-LUC vector containing REN under control of the 35S promoter was used as a positive control.

Following plasmid construction, these constructs were introduced into *Agrobacterium tumefaciens* strain GV3101, which harbored the pSoup plasmid. Subsequently, *A. tumefaciens* containing different combinations of effector and reporter plasmids was infiltrated into *N. benthamiana* plants with six to eight young leaves. After a 3-day incubation period, the ratios of LUC to REN were quantified using the Bio-Lite Luciferase Assay System (DD1201, Vazyme Biotech Co., Ltd., Nanjing, China). Images capturing LUC signals were acquired using a CCD camera (Night Shade LB 985, Germany). Primer sequences are listed in Table S10.

### Statistical analysis

Statistical analyses of data were conducted using IBM SPSS Statistics, while graphical representations were created using GraphPad Prism 8.0.1. Paired data comparisons were assessed through Student's *t*-tests (^*^P < 0.05, ^**^P < 0.01, ^***^P < 0.001). Each experiment was performed using a minimum of three biological replicates, and error bars depicted on graphs denote the standard error (SE) of the mean value. The NetWare Cloud platform (https://cloud.metware.cn) and OmicShare tools (https://www.chiplot.online/) were used for bioinformatics analyses and mapping.

## Acknowledgements

This work was supported by the Consult of Flower Industry of Jinning District (202204BI090022), General Project of Shenzhen Science and Technology and Innovation Commission (Grant No. 6020330006K0).

## Author Contributions

ZX, MN conceived and designed the experiments. RH and YW conducted the experiments. RH, YW, ZX analyzed the data. LY, JW, QX, CP, XT, GJ and MN performed the research. RH, SM and ZX wrote the manuscript. All authors read and approved the manuscript. RH and YW contributed equally to this work.

## Data availability

The datasets generated and analyzed during the current study are available in the Biological Research Project Data (BioProject), National Center for Biotechnology Information (NCBI) repository, accession: PRJNA1030783.

## Conflict of interest statement

The authors declare that they have no competing interests.

## Supplementary Material

Web_Material_uhae068
