# Supplementary material for: Multi-omics analysis reveals key regulatory defense pathways and genes involved in salt tolerance of rose plants
Source: Hortic Res. 2024 Mar 2;11(5):uhae068. doi: 10.1093/hr/uhae068 (PMC11079482; doi:10.1093/hr/uhae068)
Supplement: Web_Material_uhae068 [file web_material_uhae068.zip › Supplementary Figure 20240201.docx]

**Multi-omics analysis reveals key regulatory defense pathways and genes involved in salt tolerance of rose plants**

**Supplementary Figures and Legends**


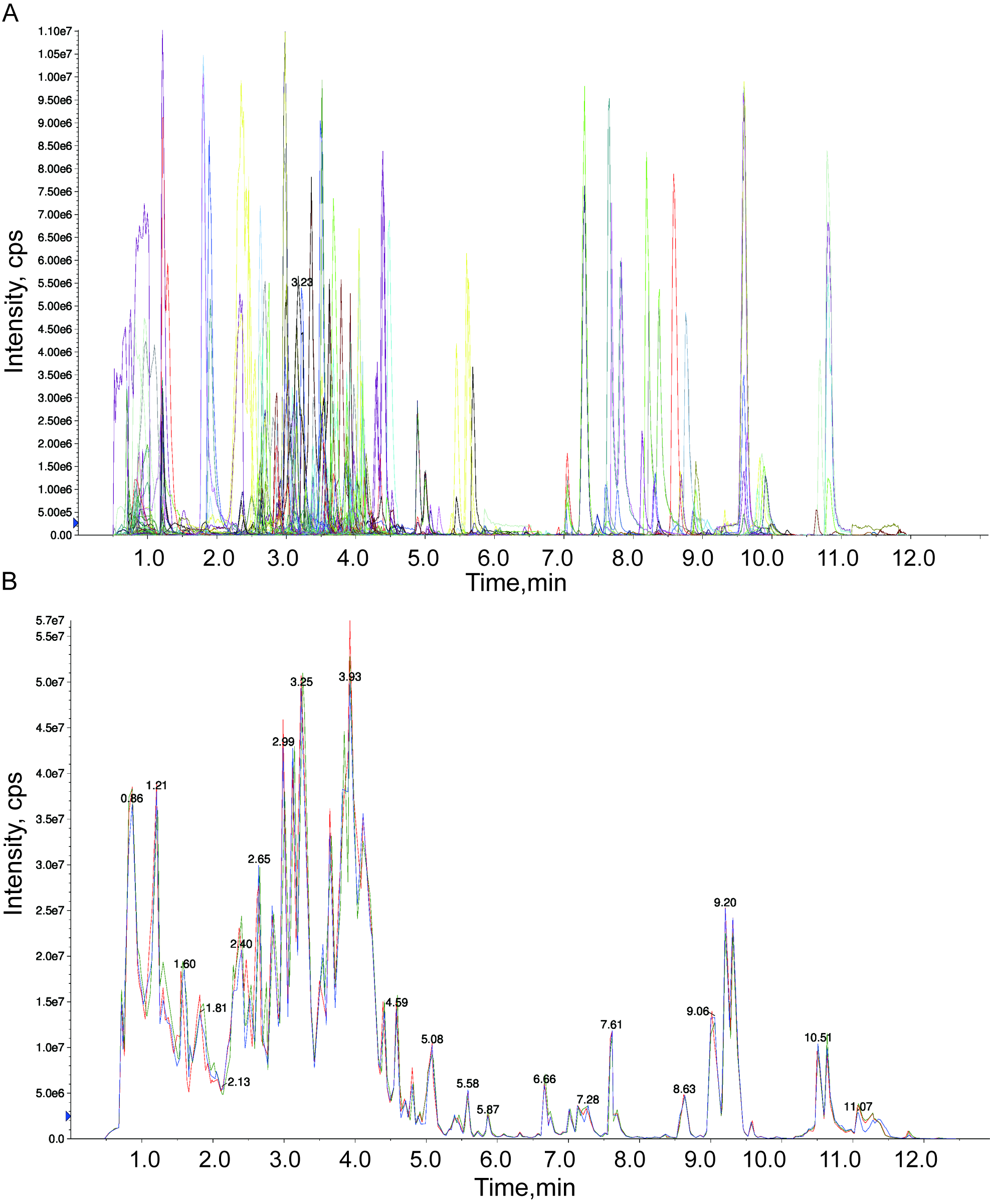


**Figure S1. Qualitative and quantitative analysis of metabolites in rose under salt stress.** (A) MRM metabolite detection multi-peak graph displaying the substances detected in JDG and DMS, with each differently colored mass spectrum peak representing a different metabolite. (B) TIC overlap diagram of the QC sample. The X-axis represents the retention time for metabolite detection, and the Y-axis represents the current intensity of the detected ion (intensity unit: cps, counts per second).


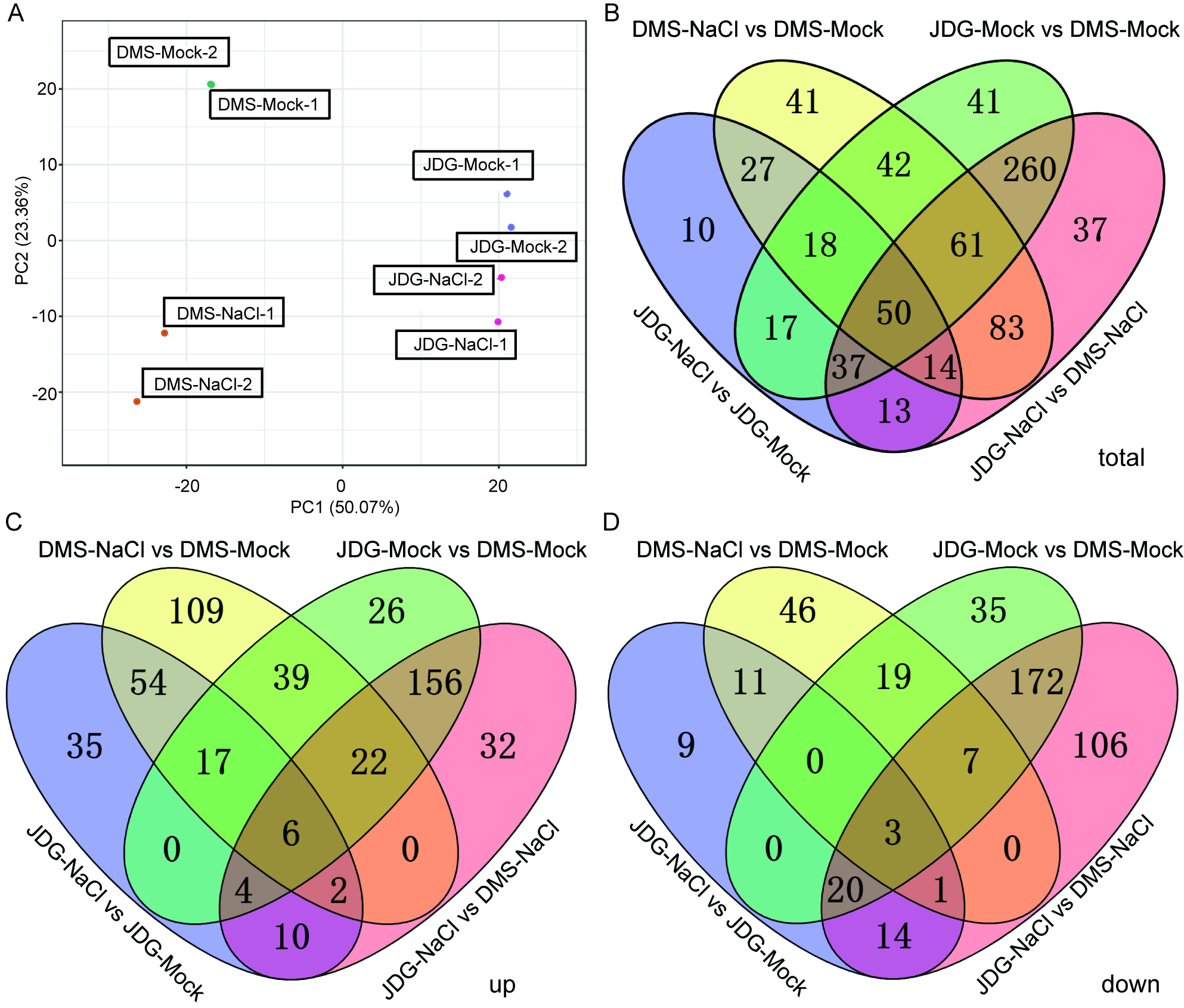


**Figure S2. Metabolomic analysis of rose under salt stress.** PCA score chart (A) and Venn diagram analysis of total metabolites (B), upregulated metabolites (C), and downregulated metabolites (D) in JDG and DMS under salt treatment.


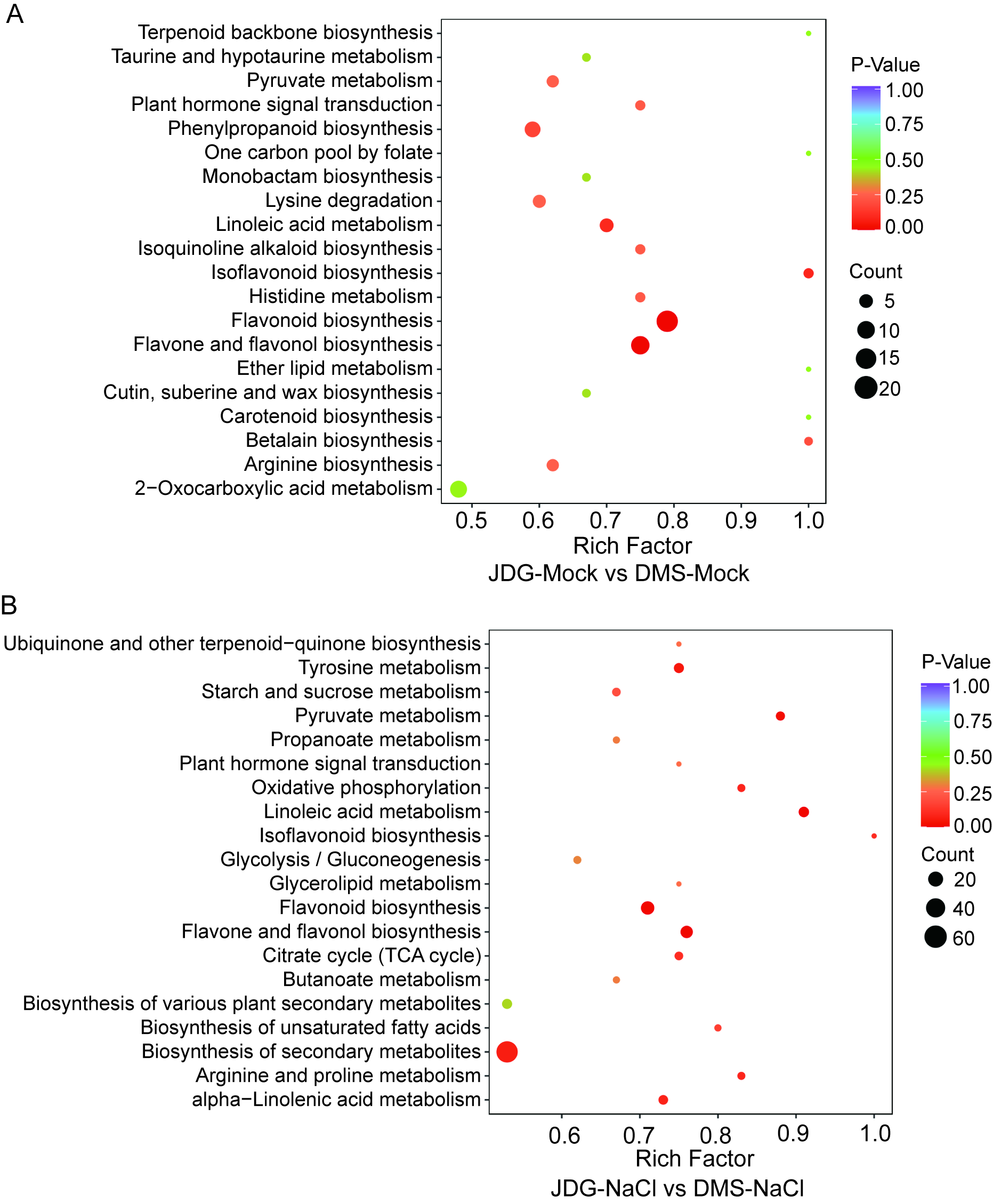


**Figure S3.** **KEGG pathway enrichment of DAMs in JDG and DMS under control and salt stress conditions**. (A) JDG-Mock vs DMS-Mock; (B) JDG-NaCl vs DMS-NaCl.


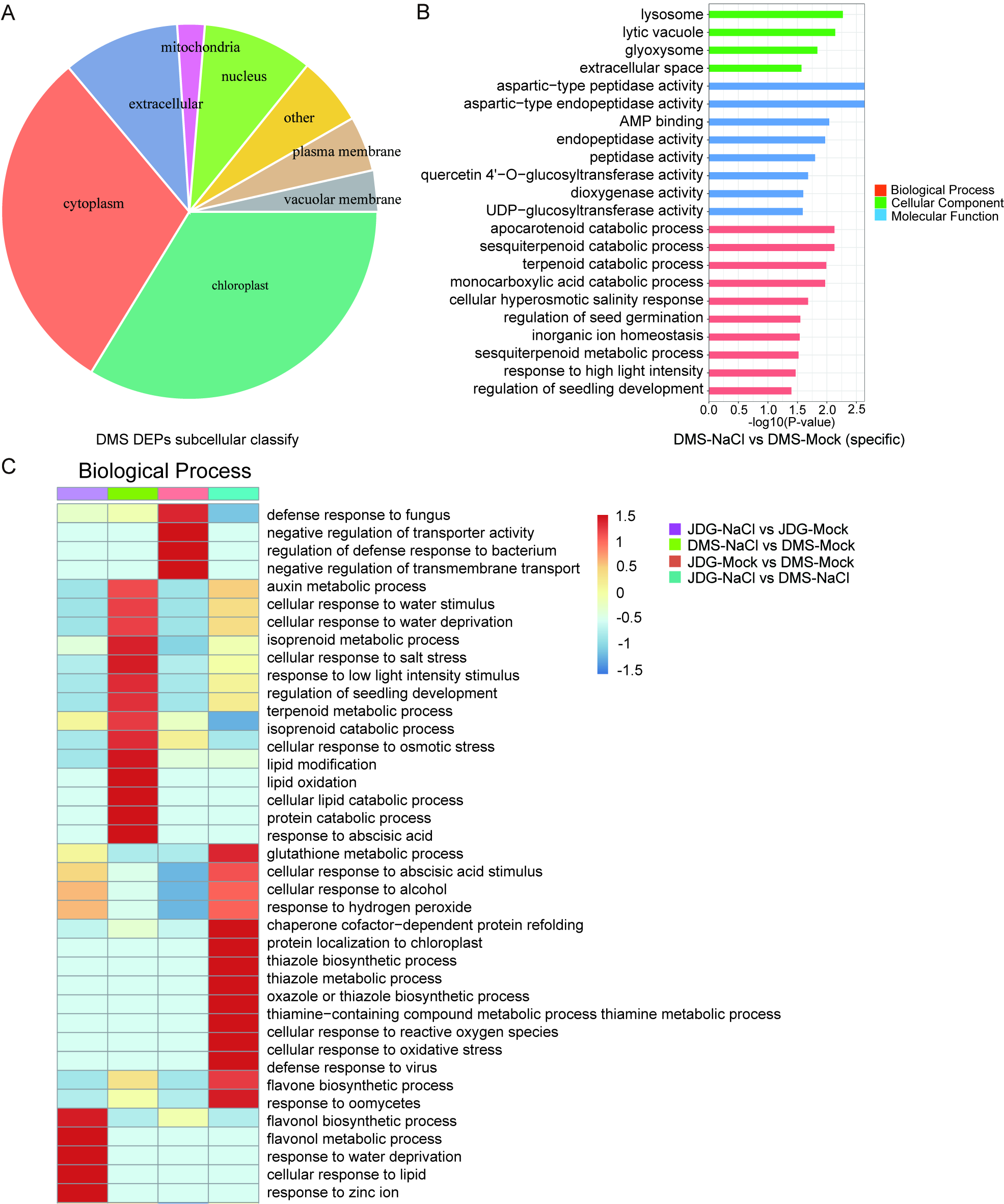


**Figure S4. Characterization of DAPs identified under salt stress.** (A) Localizations of DAPs identified in DMS. (B) Functional categorization of unique DAPs in DMS. (C) GO enrichment-based clustering analysis of all identified DAPs.


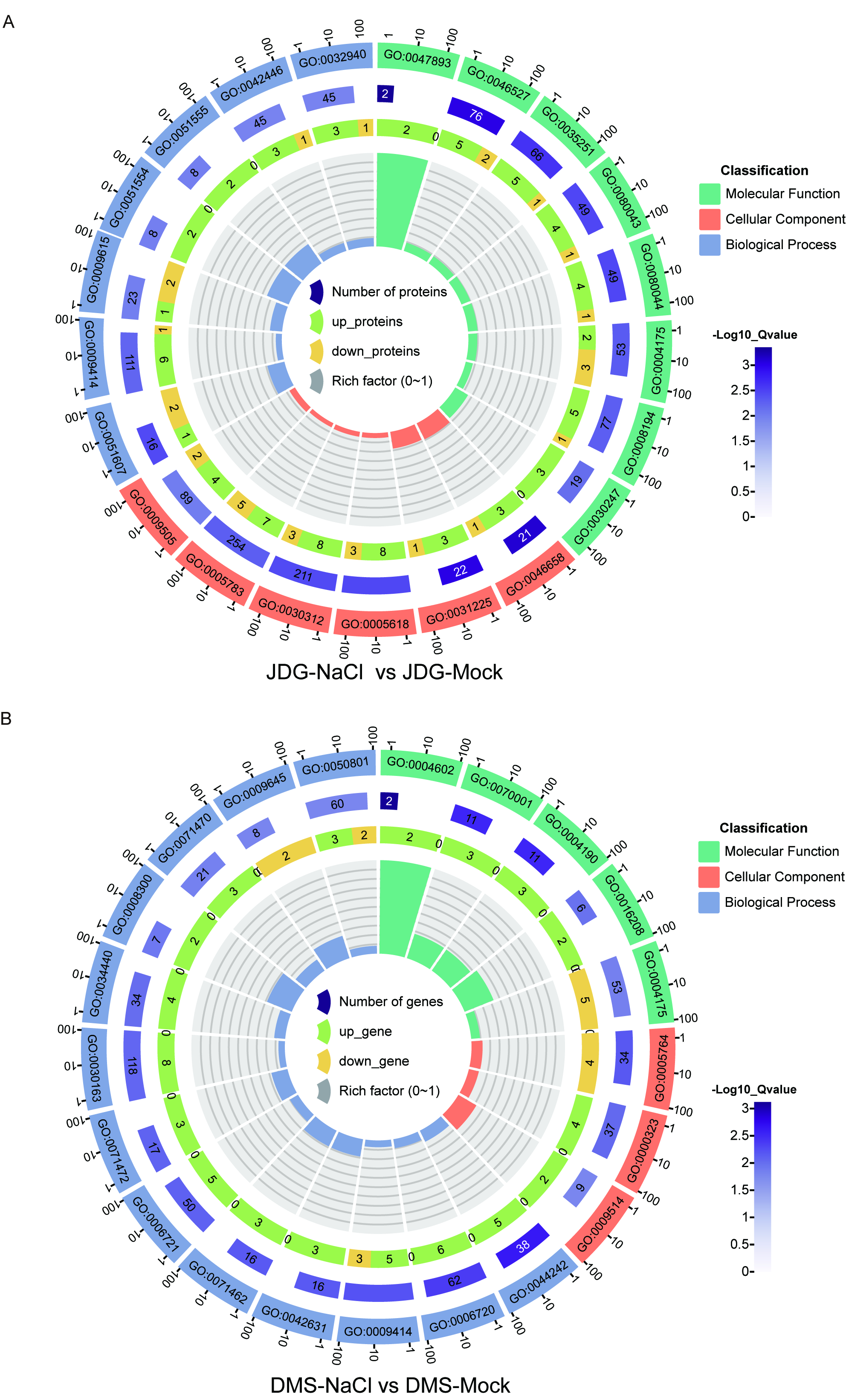


**Figure S5. GO enrichment circle diagram of DAPs.** (A) JDG-NaCl vs JDG-Mock GO enrichment circle diagram. (B) DMS-NaCl vs DMS-Mock GO enrichment circle diagram. From outside to inside, the first circle represents the classification of the enriched GO terms, with the protein quantity scale outside the circle. The second circle represents the background protein quantity. The bluer the color, the smaller the *P*-value. The third circle represents the DAPs, with green representing upregulation and yellow representing downregulation. The fourth circle represents the rich factors of each classification, and each cell represents 0.1.


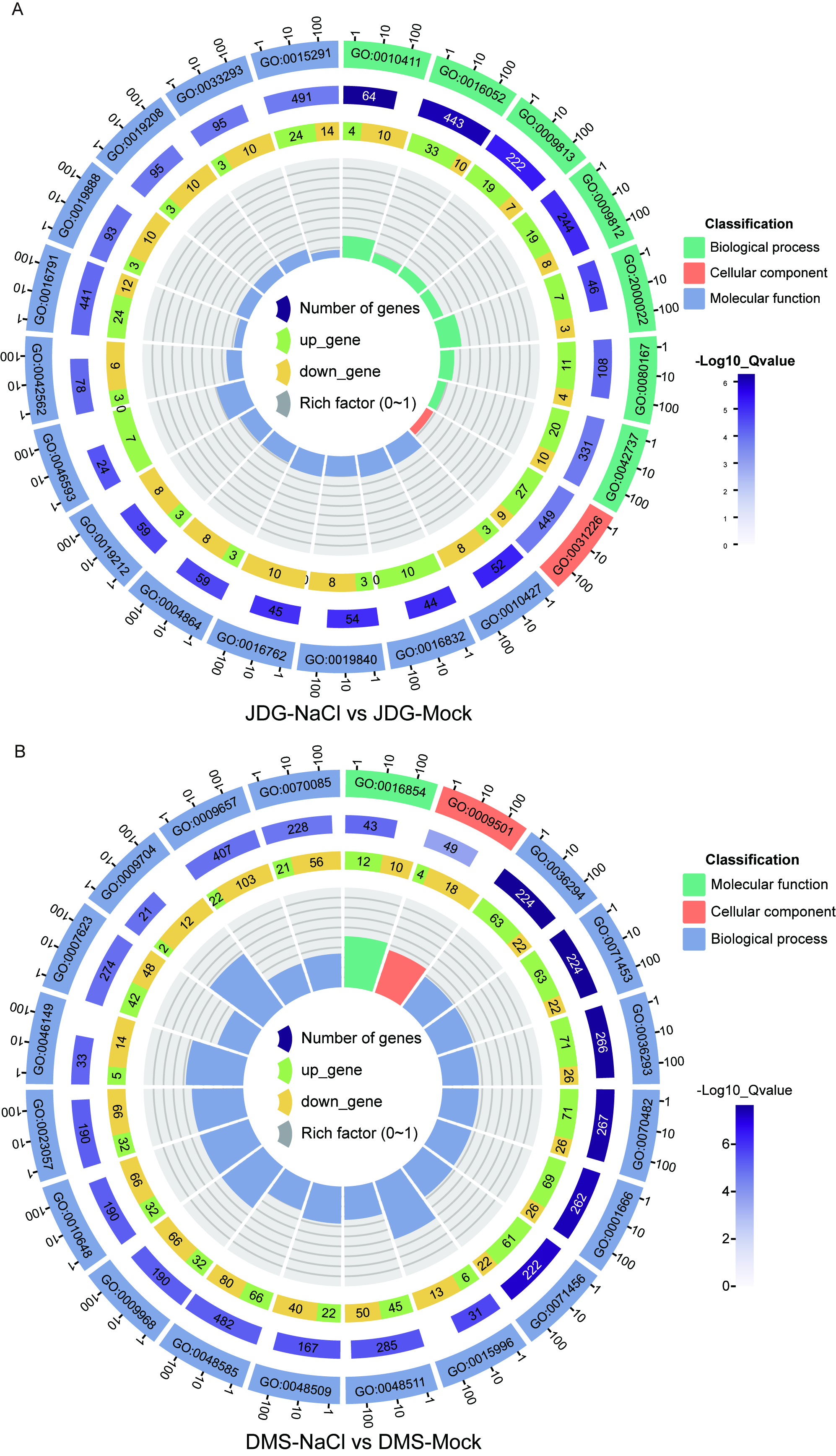


**Figure S6. GO enrichment circle diagram of DEGs.** (A) JDG-NaCl vs JDG-Mock GO enrichment circle diagram. (B) DMS-NaCl vs DMS-Mock GO enrichment circle diagram. The description of the GO enrichment circle diagram is shown in Fig. S5.


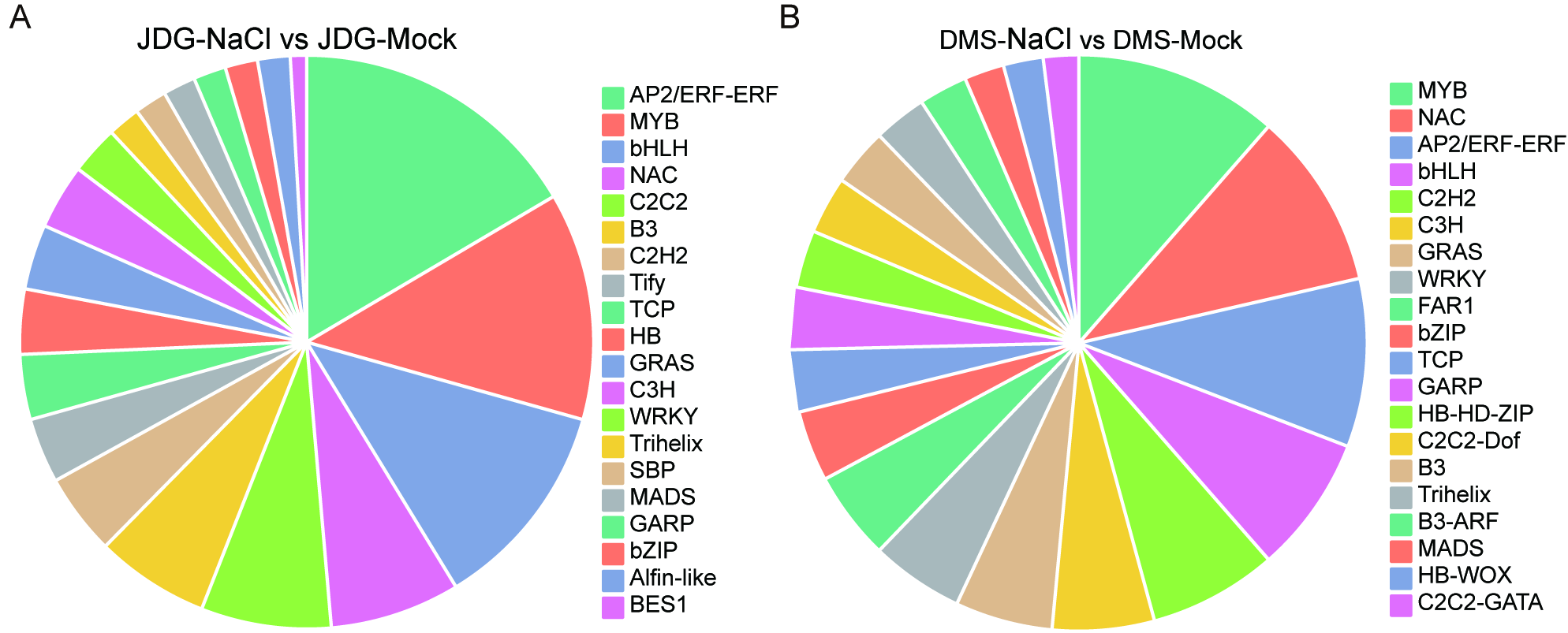


**Figure S7. Distribution of the top 20 differentially expressed transcription factors.** (A) JDG-NaCl vs JDG-Mock; (B) DMS-NaCl vs DMS-Mock.


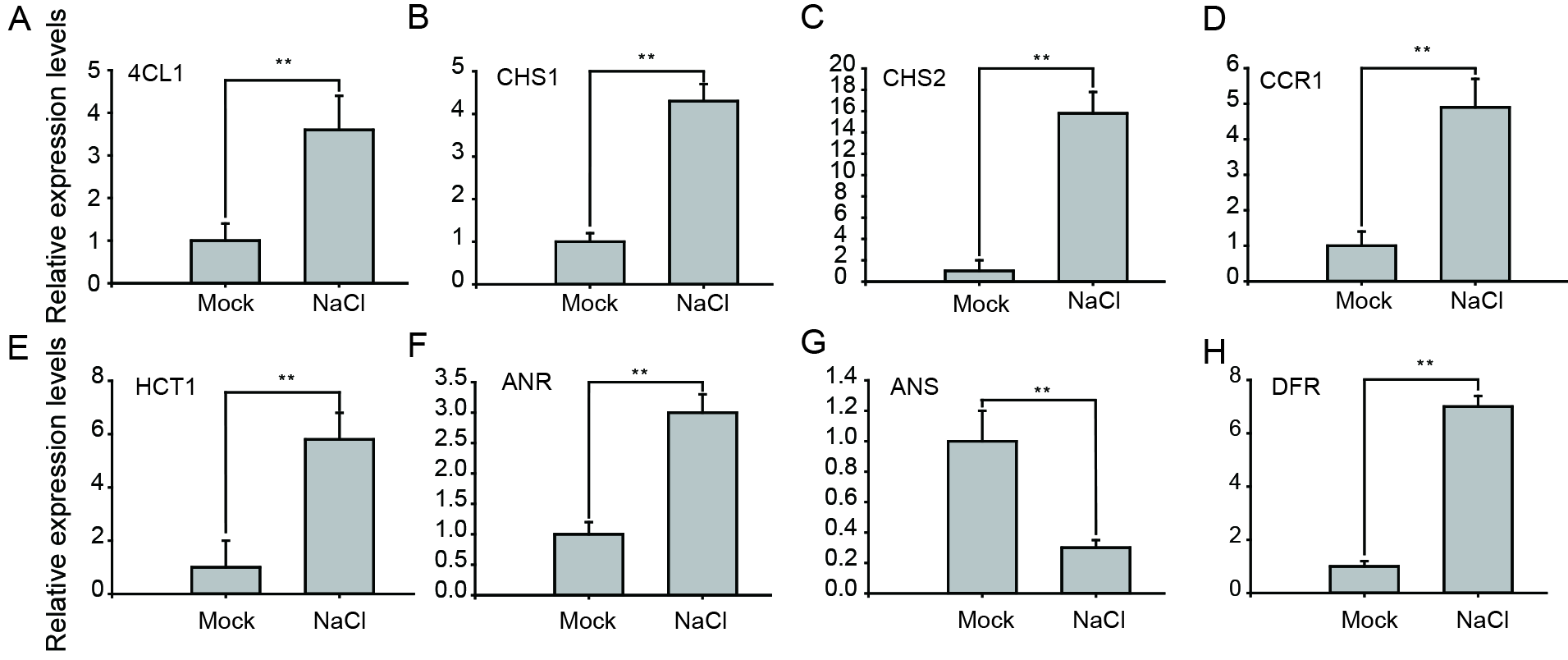


**Figure S8. Validation of transcriptome results by RT-qPCR.** (A–H) Validation of DEGs by RT-qPCR. Data are based on the mean ± SD of at least three repeated biological experiments. Significance determined using Student’s *t*-test (** *P* < 0.01).


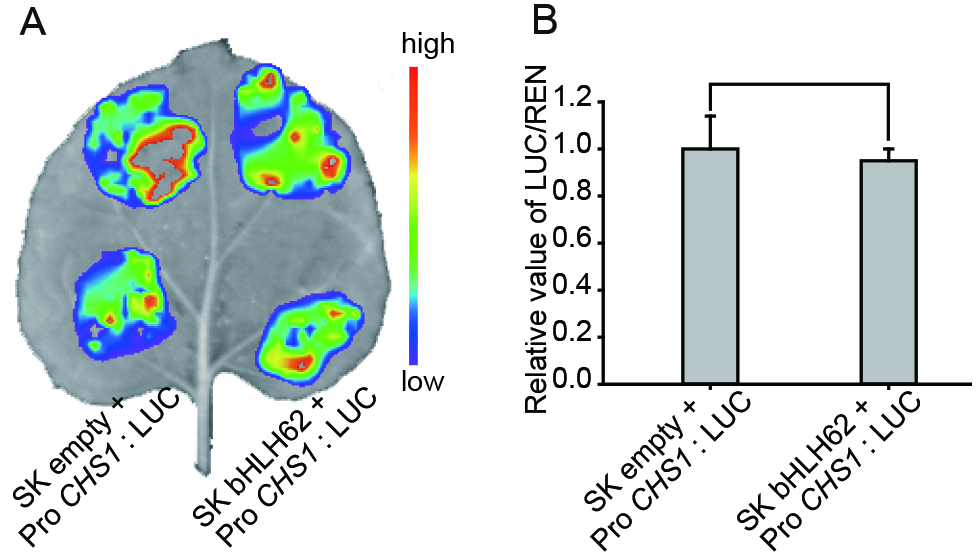


**Figure S9. bHLH62 does not inhibit the expression of *CHS1* in *Nicotiana benthamiana*** **leaves.** (A) Representative images of transient expression assays of *bHLH62* and *LUC* driven by the *CHS1* promoter in *Nicotiana benthamiana* leaves. The color scale represents the signal level. High represents a strong signal, and low represents a weak signal. (B) Relative value of LUC/REN. Data are based on the mean ± SD of at least three repeated biological experiments. Significance determined using Student’s *t*-test (** *P* < 0.01).
