# Supplementary material for: Multi-omics analysis reveals key regulatory defense pathways and genes involved in salt tolerance of rose plants
Source: Hortic Res. 2024 Mar 2;11(5):uhae068. doi: 10.1093/hr/uhae068 (PMC11079482; doi:10.1093/hr/uhae068)
Supplement: Web_Material_uhae068 [file web_material_uhae068.zip › Table S10.The primers list of genes.docx]

| **Gene** | **Primers** | **Note** |
| --- | --- | --- |
| *BHLH62* | F- ATGGAAAAGGACAGCAACTCAGCCGCACCC  R- ATGCTCAACTTTCATATGAGCTGAAGCCCC | For CDS cloning |
| *BHLH74* | F- ATG GGT AGT CAT GAC AAT GAG GAT ATG GGG  R- TTGCTCGGGTTTCATGCGGCTTGCATTTTG | For CDS cloning |
| *M13* | F-GTAAAACGACGGCCAGT  R- CAGGAAACAGCTATGAC | Vector primer For validate the construction |
| *P35S* | F- GACGTTCCAACCACGTCTTCAAAG | Vector primer For validate the construction |
| *pCHS1* | F- TGT TCA TCC AAT TAT TGT GAC ACT CGG C  R- TAC ATG GAA AAG GAG TTT GGG AAG GGA AGC GA | For promoter cloning |
| *RcGADPH-qRT* | F- GGTCAAGGTCATTGCTTGGT  R- GGATCGATCACATCGACAGA | q-PCR for reference |
| *ANR-qRT* | F1- CCCAACCCATCGTCTCAAAC  R1- TCGTCAGCTTTCCCAACTCT | q-PCR for RNA-seq validation |
| *ANS-qRT* | F1- TCTCGACGATCCCAAAGGAG  R1- CCCTACATGTCTCCCTCACC | q-PCR for RNA-seq validation |
| *DFR-qRT* | F1- CGTTCAAGGGCATTGAGGAG  R1- ACCAACGACACTGCTCTCAT | q-PCR for RNA-seq validation |
| *CHS1-qRT* | F1-AGACAAGACATGGTGGTGGT  R1- AGGCCCAAGAGCTTAGTGAG | q-PCR for RNA-seq validation |
| *CHS2-qRT* | F1- CATTGCCCGAGGTTGAGAAG  R1- GAAATCAGCCCGGGAACATC | q-PCR for RNA-seq validation |
| *HCT1-qRT* | F1- AATCACTTTCGAGGGCGTTG  R1- TCTGTATCCTCCGTGCTGTC | q-PCR for RNA-seq validation |
| *CCR1-qRT* | F1- CATGTGCTCAGACAGACAGC  R1-TTGCCAAGGCATACCACAAC | q-PCR for RNA-seq validation |
| *4CL1-qRT* | F1- TGGTGGCGGACTATGATCTC  R1- TCGTTGGAAACGGTTGCTTT | q-PCR for RNA-seq validation |

**Table S10. The primers list of genes**
